# A case of arterial switch operation with coronary elongation technique

**DOI:** 10.1186/s40792-016-0134-9

**Published:** 2016-02-03

**Authors:** Tomoyuki Matsuba, Yoshiya Shigehisa, Itsumi Imagama, Yutaka Imoto

**Affiliations:** Cardiovascular and Gastroenterological Surgery, Kagoshima University Graduate School of Medical and Dental Sciences, 8-35-1, Sakuragaoka, Kagoshima, 890-8520 Japan

**Keywords:** CHD, Great vessel anomalies, Transposition, Arterial switch

## Abstract

A 28-day-old infant with d-transposition of the great arteries underwent arterial switch operation. The coronary pattern was Yacoub type A, in which coronary transfer is usually thought to be easy. However, a dominant conus branch diverged from the proximal portion of the left coronary artery (LCA). Moreover, the LCA ostium itself was near the remote commissure in sinus 1, very far from the target re-implantation point. All of these conditions made LCA transfer very difficult. We used a coronary elongation technique to solve this problem. An inverted U-shaped flap was made in the wall of the neoaorta, and the LCA cuff was anastomosed to this flap (the inferior half from the neoaortic flap and the superior half from the LCA cuff). To prevent compression of the LCA, the neopulmonary trunk was shifted rightward. Postoperative echocardiography showed good left ventricular wall motion, and the LCA was easily visualized on chest computed tomography, with no compression from the neopulmonary artery.

## Background

Coronary artery transfer without kinking or overstretching is the key to a successful arterial switch operation (ASO). Anatomical variation of the coronary arteries, such as a single coronary orifice as in a Yacoub type B coronary pattern, with or without the intramural coronary artery, is considered a surgical risk [1–5]. However, even with a *normal* coronary artery pattern, coronary transfer can be difficult in some cases. We present herein such a case, in which the gap between the left coronary artery (LCA) orifice and the neoaorta could not be bridged with usual mobilization of the LCA because of other anatomical characteristics. ASO was successfully performed with a coronary elongation technique for the LCA.

## Case presentation

**Fig. 1:**
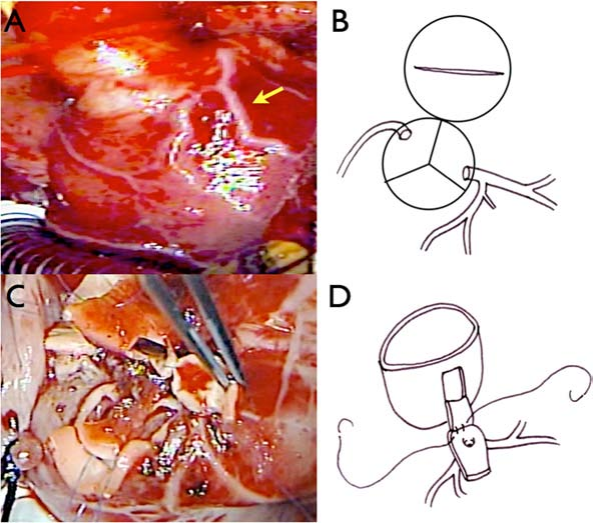
**a**, **b** A pattern of the major coronary artery was Yacoub type A, but a large conus branch diverged from the left coronary artery and supplied the right ventricular outflow tract (*arrow*). Moreover, there was the left coronary ostium near the remote commissure in sinus 1. **c**, **d** A flap was made from the neoaorta by an inverted U-shaped incision, and the LCA cuff was anastomosed with this flap by 7-0 Prolene

A 26-day-old neonate with a body weight of 3.7 kg was admitted to our hospital with diagnoses of d-transposition of the great arteries, restrictive patent foramen ovale, intact interventricular septum, patent ductus arteriosus with the use of prostaglandin E1, and pulmonary bicuspid valve. The infant’s arterial oxygen saturation was 70 %, which elevated to 90 % after balloon atrial septostomy. Coronary angiography revealed a Yacoub type A coronary pattern. At 28 days of age, the patient underwent ASO under conventional cardiopulmonary bypass with moderate hypothermia. The location of the aorta was right anterior oblique to the pulmonary artery. The coronary pattern was Yacoub type A, but a dominant conus branch diverged from the proximal LCA (Fig. 1a). An aortic cross clamp was placed, and the cold crystalloid cardioplegia was infused. After the transection of the aorta, cardiac protection was to infuse directly into the coronary ostia by a 4-Fr tube every 20 min. The left coronary ostium was seen near the remote commissure in sinus 1 (Fig. 1b). Both coronary arteries were mobilized with surrounding cuffs. The pulmonary trunk was transected just below the bifurcation. The LCA including conus branches was dissected free as much as possible, but the distance to the neoaorta remained too great. To bridge this distance, a coronary elongation technique was adopted. An inverted U-shaped flap was made from the wall of the neoaorta; the LCA cuff was anastomosed to this flap with 7-0 Prolene (Fig. 1c, d). By this technique, we elongated the LCA about 2–3 mm. The right coronary artery was transferred to the punched-out hole in the neoaorta, according to our usual technique. The pulmonary artery was reconstructed with the Lecompte maneuver. To avoid LCA compression by the neopulmonary artery, the anastomosis of the pulmonary trunk was shifted to the right. The orifice of the distal pulmonary artery was extended to the right, and most of the original orifice was over-sewn. A fresh autologous pericardial patch was used to cover the coronary cuff defects. The cardiopulmonary bypass time was 306 min, and the aorta cross-clamp time was 207 min.

**Fig. 2:**
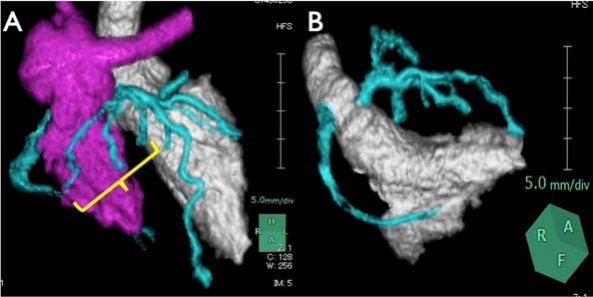
**a**, **b** Three-dimensional CT coronary angiography showed the conus branch salvaged (*square bracket*) and enough space between the left coronary artery and the left pulmonary artery. The elongated left main trunk was very smooth without stenosis

The patient’s postoperative course was uneventful. Postoperative echocardiography showed good left ventricular wall motion without dyskinesis. Computed tomography clearly revealed both coronary arteries including conus branches, with no compression of the LCA by the pulmonary artery (Fig. 2).

We were able to fill the gap between the LCA ostium and the neoaorta with the use of a coronary elongation technique. This maneuver was first performed in coronary transfer in Bland-White-Garland syndrome [2] and was later used in ASO [3, 4]. Compared to the usual trapdoor technique [5], this technique maximizes elongation, because vascular tissues extend from both sides of the anastomosis: the inferior half from the neoaortic flap and the superior half from the LCA cuff. Although lengthening of the LCA was achieved with this method, the course of the left main trunk was rather straight and ran near the neopulmonary trunk. Thus, the reconstructed LCA segment seemed vulnerable to compression by the pulmonary trunk. Therefore, we shifted the neopulmonary trunk to the right. Careful follow-up is needed to assess the status of the reconstructed coronary artery.

## Conclusions

Difficulty with coronary transfer in ASO may be encountered even in patients with a normal coronary artery pattern because of the positional relationship between the coronary ostium and the neoaorta. Coronary elongation techniques can be useful in such cases.

## Consent

Written informed consent was obtained from the patient for publication of this case report and any accompanying images. A copy of the written consent is available for review by the Editor-in-Chief of this journal.
